# Supplementary material for: Symptomatic and asymptomatic enteric protozoan parasitic infection and their association with subsequent growth parameters in under five children in South Asia and sub-Saharan Africa
Source: PLoS Negl Trop Dis. 2023 Oct 10;17(10):e0011687. doi: 10.1371/journal.pntd.0011687 (PMC10588856; doi:10.1371/journal.pntd.0011687)
Supplement: S1 Table — (DOCX) [file pntd.0011687.s001.docx]

**Supplementary table 1.** Baseline characteristics of the asymptomatic children having stool positive for enteric protozoan parasites (*Cryptosporidium, Giardia*, and *Entamoeba* *histolytica*) in seven sites of GEMS

| **Characteristics** | | ***Cryptosporidium* (+)**  n=843 (%) | ***Cryptosporidium* (-)**  n= 12,285 (%) | ***P value*** | ***Giardia* (+)**  n=3469 (%) | ***Giardia* (-)**  n=9658 | ***P value*** | ***Entamoeba histolytica* (+)**  n=299 (%) | ***Entamoeba histolytica* (-)**  n=12,829 (%) | ***P value*** |
| --- | --- | --- | --- | --- | --- | --- | --- | --- | --- | --- |
| Age group | |  |  |  |  |  |  |  |  |  |
|  | 0-11m | 347 (41.2) | 4,530 (36.9) |  | 585 (16.9) | 4,292 (44.4) |  | 132 (44.2) | 4,745 (36.9) |  |
|  | 12-23m | 276 (32.7) | 4,105 (33.4) | 0.30 | 1422 (40.9) | 2,959 (30.6) | <0.001 | 98 (32.8) | 4,283 (33.4) | 0.12 |
|  | 24-59m | 220 (26.1) | 3,650 (39.7) | 0.54 | 1463 (42.2) | 2,407 (24.9) | <0.001 | 69 (23.1) | 3,801 (29.6) | <0.001 |
| Gender (Girl) | | 366 (43.4) | 5,284 (43.0) | 0.90 | 1517 (43.7) | 4,133 (42.8) | 0.48 | 123 (41.1) | 5,527 (43.1) | 0.52 |
| Baseline Anthropometry | |  |  |  |  |  |  |  |  |  |
|  | HAZ/LAZ^¶^ | -1.39 ± 1.31 | -1.34 ± 1.31 | 0.24 | -1.54 ± 1.32 | -1.27 ± 1.30 | <0.001 | -1.51 ± 1.27 | -1.34 ± 1.31 | 0.02 |
|  | WAZ^¶^ | -1.23 ± 1.36 | -1.07± 1.31 | <0.001 | -1.19 ± 1.25 | -1.04 ± 1.34 | <0.001 | -1.05 ± 1.25 | -1.08 ± 1.32 | 0.69 |
|  | WHZ^¶^ | -0.63 ± 1.51 | -0.46 ± 1.42 | <0.001 | -0.52 ± 1.35 | -0.45 ± 1.46 | 0.02 | -0.27 ± 1.34 | -0.47 ± 1.43 | 0.01 |
| Breastfeeding status (< 2 children) | |  |  |  |  |  |  |  |  |  |
|  | Non-breastfed | 207 (24.6) | 3,883 (31.6) |  | 1,520 (43.8) | 2,570 (26.6) |  | 75 (25.1) | 4,015 (31.3) |  |
|  | Breastfed | 636 (75.4) | 8402 (68.4) | 0.13 | 1950 (56.2) | 7,088 (73.4) | <0.001 | 224 (74.9) | 8,814 (68.7) | 0.001 |
| Primary caretaker’s education | |  |  |  |  |  |  |  |  |  |
|  | Literate | 514 (61.2) | 7,420 (60.5) |  | 1843 (53.3) | 6,091 (63.2) |  | 205 (68.8) | 7,729 (60.4) |  |
|  | Illiterate | 326 (38.8) | 4,842 (39.5) | 0.83 | 1615 (46.7) | 3,553 (36.8) | 0.01 | 93 (31.2) | 5,075 (39.6) | 0.39 |
| Wealth index | |  |  |  |  |  |  |  |  |  |
|  | Poorest | 169 (20.1) | 2,341 (19.1) |  | 724 (20.9) | 1,786 (18.5) |  | 71 (23.8) | 2,439 (19.0) |  |
|  | lower middle | 149 (17.7) | 2,441 (19.9) | 0.16 | 719 (20.7) | 1,871 (19.4) | 0.37 | 50 (16.7) | 2,540 (19.8) | 0.01 |
|  | Middle | 195 (23.1) | 2,639 (21.5) | 0.83 | 735 (21.2) | 2,099 (21.7) | 0.002 | 73 (24.4) | 2,761 (21.5) | 0.73 |
|  | Upper middle | 173 (20.5) | 2,349 (19.1) | 0.83 | 658 (18.9) | 1,864 (19.3) | 0.01 | 51 (17.1) | 2,471 (19.3) | 0.26 |
|  | Richest | 157 (18.6) | 2,514 (20.5) | 0.06 | 633 (18.3) | 2,038 (21.1) | 0.01 | 54 (18.1) | 2,617 (20.4) | 0.15 |
| Drinking water | |  |  |  |  |  |  |  |  |  |
|  | Tube well water | 140 (16.6) | 2,854 (23.2) |  | 529 (15.2) | 2,465 (25.5) |  | 79 (26.4) | 2,915 (22.7) |  |
|  | Non-tube well water | 703 (83.4) | 9,431 (76.8) | 0.06 | 2941 (84.8) | 7,193 (74.5) | 0.02 | 220 (73.6) | 9,914 (77.3) | 0.66 |
| Handwashing material | |  |  |  |  |  |  |  |  |  |
|  | With soap and water | 581 (69.0) | 9,180 (74.7) |  | 2515 (72.5) | 7,246 (75.0) |  | 207 (69.2) | 9,554 (74.5) |  |
|  | Without soap | 261 (31.0) | 3,104 (25.3) | 0.09 | 954 (27.5) | 2,411 (25.0) | 0.09 | 92 (30.8) | 3,273 (25.5) | 0.23 |
| Handwashing practice | |  |  |  |  |  |  |  |  |  |
|  | Before nursing a child | 379 (44.9) | 4,753 (38.7) | 0.22 | 1355 (39.1) | 3,777 (39.1) | 0.98 | 149 (49.8) | 4,983 (38.4) | 0.21 |
|  | After cleaning a child who defecated | 447 (53.0) | 5,741 (46.7) | 0.06 | 1699 (48.9) | 4,489 (46.5) | 0.19 | 140 (46.8) | 6,048 (47.1) | 0.96 |
| Available toilet facility | |  |  |  |  |  |  |  |  |  |
|  | Sanitary/ semi-sanitary | 793 (94.1) | 11,495 (93.6) |  | 3270 (94.2) | 9,018 (93.4) |  | 286 (95.7) | 12,002 (93.6) |  |
|  | Non-sanitary | 50 (5.9) | 790 (6.4) | 0.42 | 200 (5.8) | 640 (6.6) | 0.34 | 13 (4.4) | 827 (6.5) | 0.46 |
| Co-pathogens isolated | |  |  |  |  |  |  |  |  |  |
|  | ETEC | 55 (6.5) | 920 (7.5) | 0.30 | 273 (7.9) | 702 (7.3) | 0.25 | 24 (8.0) | 951 (7.4) | 0.69 |
|  | *Campylobacter* | 107 (12.7) | 1,454 (11.8) | 0.46 | 377 (10.9) | 1,184 (12.3) | 0.03 | 30 (10.0) | 1,531 (11.9) | 0.32 |
|  | EAEC | 157 (18.6) | 2,497 (20.3) | 0.23 | 593 (17.1) | 2,061 (21.3) | <0.001 | 74 (24.8) | 2,580 (20.1) | 0.05 |
|  | Rotavirus | 30 (3.6) | 479 (3.9) | 0.62 | 123 (3.5) | 386 (4.0) | 0.24 | 38 (12.7) | 471 (3.7) | <0.001 |
|  | *Shigella* | 19 (2.3) | 212 (1.7) | 0.26 | 70 (2.0) | 161 (1.7) | 0.18920 | 3 (1.0) | 228 (1.8) | 0.31 |

^¶^ mean± SD (standard deviation); ETEC: Enterotoxigenic *E. coli*; EAEC: Enteroaggregative *E. coli;* HAZ/LAZ: height/length-for-age, WAZ: weight-for-age, and WHZ: weight-for-height z-scores; Breastfed (both exclusive and partially breastfeed
