# Supplementary material for: Symptomatic and asymptomatic enteric protozoan parasitic infection and their association with subsequent growth parameters in under five children in South Asia and sub-Saharan Africa
Source: PLoS Negl Trop Dis. 2023 Oct 10;17(10):e0011687. doi: 10.1371/journal.pntd.0011687 (PMC10588856; doi:10.1371/journal.pntd.0011687)
Supplement: S2 Table — (DOCX) [file pntd.0011687.s002.docx]

**Supplementary table 2.** Baseline characteristics of the symptomatic MSD children having stool positive for enteric protozoan parasites (*Cryptosporidium, Giardia*, and *Entamoeba histolytica*) in seven sites of GEMS

| **Characteristics** | | ***Cryptosporidium* (+)**  n=1123 (%) | ***Cryptosporidium (-)***  n= 8315 (%) | ***P-value*** | ***Giardia* (+)**  n=1786 (%) | ***Giardia (-)***  n= 7652 (%) | ***P value*** | ***Entamoeba histolytica* (+)**  n=279 (%) | ***Entamoeba histolytica* (-)**  n=9159 (%) | ***P value*** |
| --- | --- | --- | --- | --- | --- | --- | --- | --- | --- | --- |
| Age group | |  |  |  |  |  |  |  |  |  |
|  | 0-11m | 624 (55.6) | 3,405 (40.9) |  | 377 (21.1) | 3,652 (47.7) |  | 115 (41.2) | 3,914 (42.7) |  |
|  | 12-23m | 374 (33.3) | 2,830 (34.0) | 0.001 | 693 (38.8) | 2,511 (32.8) | <0.001 | 100 (35.8) | 3,104 (33.9) | 0.42 |
|  | 24-59m | 125 (11.1) | 2,080 (25.0) | <0.001 | 716(40. 1) | 1,489 (19.5) | <0.001 | 64 (22.9) | 2,141 (23.4) | 0.93 |
| Gender (Girl) | | 477 (42.5) | 3,618 (43.5) | 0.17 | 823 (46.1) | 3,272 (42.8) | 0.001 | 118 (42.3) | 3,977 (43.4) | 0.67 |
| Baseline Anthropometry | |  |  |  |  |  |  |  |  |  |
|  | HAZ/LAZ^¶^ | -1.40 ± 1.30 | -1.33 ± 1.37 | 0.93 | -1.45 ± 1.32 | -1.31 ± 1.38 | <0.001 | -1.33 ± 1.40 | -1.34 ± 1.36 | 0.93 |
|  | WAZ^¶^ | -1.70 ± 1.42 | -1.49 ± 1.38 | 0.46 | -1.53 ± 1.32 | -1.51 ± 1.41 | 0.43 | -1.45 ± 1.42 | -1.51 ± 1.39 | 0.46 |
|  | WHZ^¶^ | -1.26 ± 1.55 | -1.04 ± 1.48 | 0.23 | -1.06 ± 1.47 | -1.06 ± 1.49 | 0.96 | -0.96 ± 1.43 | -1.07 ± 1.49 | 0.23 |
| Breastfeeding status (< 24 months children) | |  |  |  |  |  |  |  |  |  |
|  | Non- breastfed | 211 (18.8) | 2,487 (29.9) |  | 817 (45.7) | 1,881 (24.6) |  | 71 (25.5) | 2,627 (28.7) |  |
|  | Breastfed | 911 (81.2) | 5,828 (70.1) | 0.001 | 969 (54.3) | 5,770 (75.4) | <0.001 | 208 (74.6) | 6,531 (71.3) | 0.53 |
| Clinical features | |  |  |  |  |  |  |  |  |  |
|  | Dysentery | 153 (13.6) | 2,084 (25.1) | <0.001 | 357 (19.9) | 1,880 (24.6) | <0.001 | 118 (42.3) | 2,119 (23.1) | <0.001 |
|  | Fever | 675 (60.1) | 5,173 (62.2) | 0.17 | 1060 (59.4) | 4,788 (62.6) | 0.01 | 155 (55.6) | 5,693 (62.2) | 0.03 |
|  | vomiting | 469 (41.8) | 3,176 (38.2) | 0.02 | 572 (32.0) | 3,073 (40.2) | <0.001 | 95 (34.1) | 3,550 (38.8) | 0.11 |
| Primary caretaker’s education | |  |  |  |  |  |  |  |  |  |
|  | Literate | 622 (55.6) | 4,762 (57.5) |  | 944 (53.0) | 4,440 (58.3) |  | 189 (68.2) | 5,195 (56.9) |  |
|  | Illiterate | 496 (44.4) | 3,520 (42.5) | 0.48 | 837 (47.0) | 3,179 (41.7) | 0.25 | 88 (31.8) | 3,928 (43.1) | 0.18 |
| Wealth index | |  |  |  |  |  |  |  |  |  |
|  | Poorest | 256 (22.8) | 1,770 (21.3) |  | 417 (23.4) | 1,609 (21.0) |  | 43 (15.4) | 1,983 (21.7) |  |
|  | lower middle | 197 (17.5) | 1,616 (19.5) | 0.04 | 332 (18.6) | 1,481 (19.4) | <0.001 | 61 (21.9) | 1,752 (19.1) | 0.08 |
|  | Middle | 247 (21.9) | 1,746 (21.0) | 0.81 | 359 (20.1) | 1,634 (21.4) | <0.001 | 59 (21.2) | 1,934 (21.1) | 0.15 |
|  | Upper middle | 213 (18.9) | 1,566 (18.9) | 0.62 | 345 (19.3) | 1,434 (18.8) | 0.57 | 64 (22.9) | 1,715 (18.7) | 0.002 |
|  | Richest | 210 (18.7) | 1,611 (19.4) | 0.21 | 333 (18.7) | 1,488 (19.5) | 0.14 | 52 (18.6) | 1,769 (19.3) | 0.19 |
| Drinking water | |  |  |  |  |  |  |  |  |  |
|  | Tube well water | 135 (12.0) | 1,540 (18.5) |  | 161 (9.0) | 1,514 (19.8) |  | 91 (32.6) | 1,584 (17.3) |  |
|  | Non-tube well water | 988 (87.9) | 6,775 (81.5) | 0.01 | 1625 (90.9) | 6,138 (80.2) | 0.003 | 188 (67.4) | 7,575 (82.7) | 0.04 |
| Handwashing material | |  |  |  |  |  |  |  |  |  |
|  | With soap and water | 810 (72.1) | 6,319 (76.0) |  | 1323 (74.1) | 5,806 (75.9) |  | 209 (74.9) | 6,920 (75.6) |  |
|  | Without soap | 313 (27.9) | 1,995 (24.0) | 0.001 | 463 (25.9) | 1,845 (24.1) | 0.34 | 70 (25.1) | 2,238 (24.4) | 0.92 |
| Handwashing practice | |  |  |  |  |  |  |  |  |  |
|  | Before nursing a child | 485 (43.2) | 3,198 (38.5) | 0.16 | 646 (36.2) | 3,037 (39.7) | 0.19 | 103 (36.9) | 3,580 (39.1) | 0.61 |
|  | After cleaning a child who defecated | 537 (47.8) | 3,711 (44.6) | 0.29 | 761 (42.6) | 3,487 (45.6) | 0.21 | 109 (39.1) | 4,139 (45.2) | 0.33 |
| Improved toilet facility | |  |  |  |  |  |  |  |  |  |
|  | Sanitary/ semi-sanitary | 1071 (95.4) | 7,906 (95.1) |  | 1697 (95.0) | 7,280 (95.1) |  | 268 (96.1) | 8,709 (95.1) |  |
|  | Non-sanitary | 52 (4.6) | 409 (4.9) | 0.54 | 89 (4.9) | 372 (4.9) | 0.88 | 11 (3.9) | 450 (4.9) | 0.72 |
| Co-pathogens isolated | |  |  |  |  |  |  |  |  |  |
|  | ETEC | 124 (11.0) | 943 (11.3) | 0.77 | 205 (11.5) | 862 (11.3) | 0.80 | 14 (5.0) | 1,053 (11.5) | 0.001 |
|  | *Campylobacter* | 149 (13.3) | 1,022 (12.3) | 0.35 | 211 (11.8) | 960 (12.6) | 0.40 | 38 (13.6) | 1,133 (12.4) | 0.53 |
|  | EAEC | 220 (19.6) | 1,626 (19.6) | 0.98 | 258 (14.5) | 1,588 (20.8) | <0.001 | 61 (21.9) | 1,785 (19.5) | 0.33 |
|  | Rotavirus | 161 (14.3) | 1,586 (19.1) | <0.001 | 208 (11.7) | 1,539 (20.1) | <0.001 | 65 (23.3) | 1,682 (18.4) | 0.04 |
|  | *Shigella* | 69 (6.1) | 1,041 (12.5) | <0.001 | 176 (9.9) | 934 (12.2) | 0.01 | 55 (19.7) | 1,055 (11.5) | <0.001 |

^¶^ mean± SD (standard deviation); ETEC: Enterotoxigenic *E. coli*; EAEC: Enteroaggregative *E. coli;* HAZ/LAZ: height/length-for-age, WAZ: weight-for-age, and WHZ: weight-for-height z-scores; Breastfeed (both exclusive and partially breastfed)
