# Supplementary material for: Symptomatic and asymptomatic enteric protozoan parasitic infection and their association with subsequent growth parameters in under five children in South Asia and sub-Saharan Africa
Source: PLoS Negl Trop Dis. 2023 Oct 10;17(10):e0011687. doi: 10.1371/journal.pntd.0011687 (PMC10588856; doi:10.1371/journal.pntd.0011687)
Supplement: S3 Table — (DOCX) [file pntd.0011687.s003.docx]

**Supplementary Table 3.** Children having more than one enteric protozoan parasite

| **Stool positive for the parasite** | **n= 22,566 (%)** |
| --- | --- |
| No parasite | 15,456 (68.5) |
| At least one parasite | 6,434 (28.5) |
| At least two parasites | 662 (2.9) |
| Three Parasites | 14 (0.06) |
