# Supplementary material for: Symptomatic and asymptomatic enteric protozoan parasitic infection and their association with subsequent growth parameters in under five children in South Asia and sub-Saharan Africa
Source: PLoS Negl Trop Dis. 2023 Oct 10;17(10):e0011687. doi: 10.1371/journal.pntd.0011687 (PMC10588856; doi:10.1371/journal.pntd.0011687)
Supplement: S4 Table — (DOCX) [file pntd.0011687.s004.docx]

**Supplementary Table 4.** Comparing enteric protozoan parasite co-infections detected among asymptomatic and symptomatic MSD children in GEMS (n=22,567)

| **Co-infections** | **Asymptomatic Children**  **n (%)** | **Symptomatic MSD children**  **n (%)** | **P value** |
| --- | --- | --- | --- |
| *Cryptosporidium* (-) & *Giardia* (-) | 3,774 (93.3) | 2,443 (91.3) | ref |
| ***Cryptosporidium* (+) & *Giardia* (+)** | 270 (6.7) | 234 (8.7) | 0.013 |
| *Cryptosporidium* (+) & *Giardia* (-) | 573 (4.4) | 889 (9.4) | <0.001 |
| *Cryptosporidium* (-) & *Giardia* (+) | 3,200 (24.4) | 1,552 (16.4) | <0.001 |
|  |  |  |  |
| *Cryptosporidium* (-) & *E. histolytica* (-) | 1,097 (97.9) | 1,310 (96.5) | ref |
| ***Cryptosporidium* (+) & *E. histolytica* (+)** | 23 (2.1) | 47 (3.4) | 0.22 |
| *Cryptosporidium* (+) & *E. histolytica* (-) | 820 (6.3) | 1,076 (11.4) | <0.001 |
| *Cryptosporidium* (-) & *E. histolytica* (+) | 276 (2.1) | 232 (2.5) | 0.51 |
|  |  |  |  |
| *Giardia* (-) & *E. histolytica* (-) | 3,612 (97.9) | 1,965 (97.5) | ref |
| ***Giardia* (+) & *E. histolytica* (+)** | 79 (2.1) | 51 (2.5) | 0.62 |
| *Giardia* (+) & *E. histolytica* (-) | 3,391 (25.8) | 1,735 (18.4) | <0.001 |
| *Giardia* (-) & *E. histolytica* (+) | 220 (1.7) | 228 (2.4) | 0.14 |

Findings from bivariate analysis and simple logistic regression after controlling for the site (country) as a cluster for children having MSD and healthy asymptomatic children; (+): positive and (-): negative

Only 3% of children had more than one parasite detected in their stool during enrollment. Around 3% (n=662) children were positive for more than two parasites in stool and less than 0.05% (n=14) children were positive for three parasites simultaneously (Supplementary Table 3). Co-infection with *Cryptosporidium* and *Giardia* (8.7%); *Cryptosporidium* and *E. histolytica* (3.4%); *Giardia* and *E. histolytica* (2.5%) were more observed among the symptomatic MSD children (Supplementary Table 4) in comparison to asymptomatic children.
