# Supplementary material for: Symptomatic and asymptomatic enteric protozoan parasitic infection and their association with subsequent growth parameters in under five children in South Asia and sub-Saharan Africa
Source: PLoS Negl Trop Dis. 2023 Oct 10;17(10):e0011687. doi: 10.1371/journal.pntd.0011687 (PMC10588856; doi:10.1371/journal.pntd.0011687)
Supplement: S5 Table — a. Age-specific association between enteric protozoan parasites co-infections and child anthropometric measurements among symptomatic MSD children on growth measures among under 5 children in South Asia and sub-Saharan Africa using multiple linear regression modeling (Dependent variables: HAZ/LAZ, WAZ, and WHZ). b. Age-specific association between enteric protozoan parasites co-infections and child anthropometric measurements among asymptomatic children on growth measures among under 5 children in South Asia and sub-Saharan Africa using multiple linear regression modeling (Dependent variables: HAZ/LAZ, WAZ, and WHZ). (DOCX) [file pntd.0011687.s005.docx]

**Supplementary Table 5 (a):** Age-specific association between enteric protozoan parasites co-infections and child anthropometric measurements among symptomatic MSD children on growth measures among under 5 children in South Asia and sub-Saharan Africa using multiple linear regression modeling (Dependent variables: HAZ/LAZ, WAZ, and WHZ)

|  | **Among the symptomatic MSD children** | | | | | |
| --- | --- | --- | --- | --- | --- | --- |
|  | **Coef. (95% CI) *** | **P value** | **Coef. (95% CI) *** | **P value** | **Coef. (95% CI) *** | **P value** |
|  | ***Crypto (+) and E. histolytica (+)*** |  | ***Crypto (+) and E. histolytica (-)*** |  | ***Crypto (-) and E. histolytica (+)*** |  |
|  | **0-11 months** | | | | | |
| **HAZ** | **0.41 (0.04, 0.79)** | **0.03** | **-0.16(-0.24, -0.08)** | **<0.001** | -0.15 (-0.34, 0.04) | 0.12 |
| **WAZ** | **0.53 (0.09, 0.97)** | **0.02** | **-0.24(-0.33, -0.14)** | **<0.001** | -0.01 (-0.23, 0.2) | 0.91 |
| **WHZ** | 0.38 (-0.11, 0.87) | 0.13 | **-0.23(-0.34, -0.13)** | **<0.001** | 0.05 (-0.19, 0.29) | 0.68 |
|  | **12-23 months** | | | | | |
| **HAZ** | -0.29 (-0.75, 0.16) | 0.21 | **-0.18 (-0.28, -0.08)** | **<0.001** | 0.08 (-0.12, 0.28) | 0.45 |
| **WAZ** | -0.43 (-0.89, 0.03) | 0.07 | **-0.27 (-0.38, -0.16)** | **<0.001** | 0.03 (-0.17, 0.24) | 0.75 |
| **WHZ** | -0.21 (-0.69, 0.28) | 0.40 | **-0.25 (-0.37, -0.14)** | **<0.001** | -0.01 (-0.23, 0.21) | 0.95 |
|  | **24-59 months** | | | | | |
| **HAZ** | -0.60 (-1.43, 0.24) | 0.16 | **-0.27 (-0.44, -0.10)** | **<0.001** | **0.32 (0.08, 0.55)** | **0.01** |
| **WAZ** | -0.19 (-0.87, 0.49) | 0.58 | **-0.19 (-0.34, -0.04)** | **0.01** | 0.15 (-0.07, 0.36) | 0.18 |
| **WHZ** | 0.15 (-0.52, 0.82) | 0.66 | -0.07 (-0.22, 0.09) | 0.40 | -0.05 (-0.28, 0.17) | 0.64 |
|  | ***Crypto (+) and Giardia (+)*** |  | ***Crypto (+) and Giardia (-)*** |  | ***Crypto (-) and Giardia (+)*** |  |
|  | **0-11 months** | | | | | |
| **HAZ** | -0.001 (-0.20,0.19) | 0.99 | **-0.15 (-0.23, -0.06)** | **<0.001** | 0.004 (-0.11, 0.12) | 0.95 |
| **WAZ** | -0.19 (-0.42, 0.05) | 0.12 | **-0.18 (-0.28, -0.09)** | **<0.001** | 0.05 (-0.07, 0.18) | 0.40 |
| **WHZ** | **-0.32 (-0.59, -0.05)** | **0.02** | **-0.16 (-0.27, -0.05)** | **<0.001** | 0.01 (-0.14, 0.15) | 0.93 |
|  | **12-23 months** | | | | | |
| **HAZ** | -0.04 (-0.23, 0.14) | 0.65 | -0.21 (-0.33, -0.10) | **<0.001** | 0.03 (-0.05, 0.12) | 0.47 |
| **WAZ** | -0.15 (-0.34, 0.05) | 0.14 | -0.31 (-0.43, -0.19) | **<0.001** | 0.09 (0.004, 0.18) | 0.04 |
| **WHZ** | -0.18 (-0.38, 0.02) | 0.08 | -0.27 (-0.39, -0.14) | **<0.001** | 0.11 (0.01, 0.20) | 0.03 |
|  | **24-59 months** | | | | | |
| **HAZ** | **-0.52 (-0.81, -0.23)** | **<0.001** | -0.18 (-0.38, 0.01) | 0.07 | 0.002 (-0.08, 0.08) | 0.97 |
| **WAZ** | **-0.27 (-0.53, -0.02)** | **0.04** | -0.16 (-0.33, 0.02) | 0.07 | 0.002 (-0.07, 0.08) | 0.97 |
| **WHZ** | 0.004 (-0.28, 0.29) | 0.98 | -0.09 (-0.27, 0.09) | 0.34 | 0.02(-0.06, 0.10) | 0.62 |
|  | ***Giardia (+) and E. histolytica (+)*** |  | ***Giardia (+) and E. histolytica (-)*** |  | ***Giardia (-) and E. histolytica (+)*** |  |
|  | **0-11 months** | | | | | |
| **HAZ** | -0.07 (-0.70, 0.57) | 0.84 | -0.02 (-0.12, 0.08) | 0.75 | -0.08 (-0.25, 0.1) | 0.40 |
| **WAZ** | 0.52 (-0.25, 1.29) | 0.19 | -0.05 (-0.16, 0.06) | 0.40 | 0.03 (-0.17, 0.23) | 0.76 |
| **WHZ** | **0.92 (0.03, 1.81)** | **0.04** | **-0.13 (-0.26, -0.01)** | **0.04** | 0.04 (-0.18, 0.26) | 0.73 |
|  | **12-23 months** | | | | | |
| **HAZ** | 0.09 (-0.31, 0.49) | 0.66 | 0.01 (-0.07, 0.09) | 0.88 | -0.003 (-0.21, 0.20) | 0.97 |
| **WAZ** | -0.16 (-0.59, 0.26) | 0.45 | 0.05 (-0.03, 0.13) | 0.24 | -0.04 (-0.26, 0.17) | 0.69 |
| **WHZ** | -0.32 (-0.76, 0.13) | 0.16 | 0.07 (-0.02, 0.15) | 0.14 | -0.01 (-0.24, 0.22) | 0.92 |
|  | **24-59 months** | | | | | |
| **HAZ** | 0.68 (0.28, 1.07) | <0.001 | -0.07 (-0.15, 0.01) | 0.10 | -0.02 (-0.29, 0.26) | 0.91 |
| **WAZ** | 0.61 (0.27, 0.96) | <0.001 | -0.05 (-0.12, 0.02) | 0.19 | -0.17 (-0.41, 0.07) | 0.17 |
| **WHZ** | 0.37 (-0.01, 0.76) | 0.06 | 0.002 (-0.08, 0.08) | 0.97 | -0.26 (-0.52, -0.01) | 0.04 |

**Supplementary Table 5 (b):** Age-specific association between enteric protozoan parasites co-infections and child anthropometric measurements among asymptomatic children on growth measures among under 5 children in South Asia and sub-Saharan Africa using multiple linear regression modeling (Dependent variables: HAZ/LAZ, WAZ, and WHZ)

|  | **Among the asymptomatic children** | | | | | |
| --- | --- | --- | --- | --- | --- | --- |
|  | **Coef. (95% CI) *** | **P value** | **Coef. (95% CI) *** | **P value** | **Coef. (95% CI) *** | **P value** |
|  | ***Crypto (+) and E. histolytica (+)*** |  | ***Crypto (+) and E. histolytica (-)*** |  | ***Crypto (-) and E. histolytica (+)*** |  |
|  | **0-11 months** | | | | | |
| **HAZ** | -0.12 (-0.81, 0.56) | 0.73 | -0.03 (-0.13, 0.08) | 0.63 | -0.10 (-0.27, 0.06) | 0.23 |
| **WAZ** | -0.37 (-1.12, 0.38) | 0.33 | **-0.15 (-0.26, -0.04)** | **0.01** | -0.01(-0.19, 0.16) | 0.89 |
| **WHZ** | -0.33 (-1.17, 0.51) | 0.44 | **-0.20 (-0.32, -0.08)** | **<0.001** | 0.10 (-0.10, 0.29) | 0.34 |
|  | **12-23 months** | | | | | |
| **HAZ** | 0.48 (-0.04, 1.00) | 0.07 | -0.06 (-0.17, 0.06) | 0.33 | 0.03 (-0.18, 0.23) | 0.79 |
| **WAZ** | -0.01(-0.53, 0.51) | 0.97 | **-0.22 (-0.34, -0.11)** | **<0.001** | -0.13 (-0.34, 0.09) | 0.24 |
| **WHZ** | -0.30 (-0.88, 0.28) | 0.32 | **-0.26 (-0.39, -0.13)** | **<0.001** | -0.18 (-0.41, 0.05) | 0.12 |
|  | **24-59 months** | | | | | |
| **HAZ** | -0.36 (-1.06, 0.34) | 0.32 | -0.09 (-0.2, 0.03) | 0.14 | -0.09 (-0.30, 0.13) | 0.42 |
| **WAZ** | -0.36 (-1.09, 0.36) | 0.33 | -0.04 (-0.14, 0.07) | 0.47 | -0.15 (-0.34, 0.04) | 0.12 |
| **WHZ** | -0.19 (-0.93, 0.55) | 0.62 | 0.03 (-0.08, 0.13) | 0.62 | -0.18 (-0.38, 0.02) | 0.07 |
|  | ***Crypto (+) and Giardia (+)*** |  | ***Crypto (+) and Giardia (-)*** |  | ***Crypto (-) and Giardia (+)*** |  |
|  | **0-11 months** | | | | | |
| **HAZ** | 0.01 (-0.24, 0.25) | 0.97 | -0.02 (-0.14, 0.09) | 0.68 | **-0.15 (-0.23, -0.06)** | **<0.001** |
| **WAZ** | -0.02 (-0.29, 0.24) | 0.85 | **-0.17 (-0.29, -0.05)** | **0.01** | **-0.14 (-0.23, -0.05)** | **<0.001** |
| **WHZ** | -0.06 (-0.35, 0.24) | 0.71 | **-0.22 (-0.35, -0.08)** | **<0.001** | **-0.14 (-0.24, -0.04)** | **0.01** |
|  | **12-23 months** | | | | | |
| **HAZ** | -0.05 (-0.24, 0.14) | 0.63 | 0.04 (-0.10, 0.18) | 0.55 | **-0.16 (-0.22, -0.10)** | **<0.001** |
| **WAZ** | -0.16 (-0.34, 0.02) | 0.08 | **-0.19 (-0.33, -0.04)** | **0.01** | **-0.07 (-0.14, -0.01)** | **0.02** |
| **WHZ** | -0.19 (-0.38, 0.01) | 0.07 | **-0.28 (-0.43, -0.12)** | **<0.001** | 0.002 (-0.06, 0.07) | 0.94 |
|  | **24-59 months** | | | | | |
| **HAZ** | -0.06 (-0.24,0.11) | 0.47 | -0.09 (-0.24, 0.07) | 0.26 | **-0.09 (-0.14, -0.03)** | **<0.001** |
| **WAZ** | -0.03 (-0.18,0.13) | 0.75 | -0.05 (-0.19, 0.09) | 0.48 | -0.02 (-0.07, 0.03) | 0.39 |
| **WHZ** | 0.02 (-0.14,0.19) | 0.76 | 0.01 (-0.13, 0.15) | 0.87 | 0.05 (-0.01, 0.1) | 0.08 |
|  | ***Giardia (+) and E. histolytica (+)*** |  | ***Giardia (+) and E. histolytica (-)*** |  | ***Giardia (-) and E. histolytica (+)*** |  |
|  | **0-11 months** | | | | | |
| **HAZ** | 0.35 (-0.08, 0.77) | 0.11 | -0.15 (-0.23, -0.07) | **<0.001** | -0.16 (-0.33, 0.02) | 0.08 |
| **WAZ** | 0.29 (-0.17, 0.74) | 0.22 | -0.15 (-0.23, -0.06) | **<0.001** | -0.06 (-0.24, 0.13) | 0.54 |
| **WHZ** | 0.15 (-0.35, 0.65) | 0.56 | **-0.14 (-0.24, -0.04)** | **0.01** | 0.09 (-0.12, 0.29) | 0.40 |
|  | **12-23 months** | | | | | |
| **HAZ** | -0.14 (-0.50, 0.22) | 0.43 | **-0.16 (-0.22, -0.10)** | **<0.001** | **0.25 (0.02, 0.48)** | **0.03** |
| **WAZ** | **-0.37 (-0.72, -0.03)** | **0.04** | **-0.09 (-0.15, -0.03)** | **0.01** | 0.003 (-0.23, 0.24) | 0.98 |
| **WHZ** | **-0.41 (-0.78, -0.03)** | **0.03** | -0.01 (-0.08, 0.05) | 0.68 | -0.15 (-0.40, 0.11) | 0.26 |
|  | **24-59 months** | | | | | |
| **HAZ** | -0.06 (-0.39, 0.27) | 0.72 | **-0.09 (-0.14, -0.04)** | **<0.001** | -0.07 (-0.33, 0.19) | 0.59 |
| **WAZ** | -0.13 (-0.42, 0.16) | 0.38 | -0.02 (-0.07, 0.03) | 0.43 | -0.15 (-0.38, 0.08) | 0.21 |
| **WHZ** | -0.19 (-0.50, 0.12) | 0.22 | **0.05 (0.003, 0.10)** | **0.04** | -0.16 (-0.40, 0.08) | 0.19 |

* Adjusted for sex, breastfeeding status, primary caretaker’s education, WASH, wealth index, co-pathogens (ETEC, EAEC, *Shigella, Campylobacter*, and Rotavirus), site, and history of comorbidity (malaria, typhoid, pneumonia, diarrhea, dysentery) at day 60 follow up. Abbreviation: Coef.: coefficient, CI: confidence interval; HAZ/LAZ: height/length-for-age, WAZ: weight-for-age, and WHZ: weight-for-height z-scores; enteric protozoan parasites were detected from the stool sample during enrollment; Anthropometric measurements were taken during enrollment and after 60 days of enrollment (during the follow-up visit); Separate models were performed to see the association of enteric protozoan parasites infection with a child’s height-for-age, weight-for-age, and weight-for-height z-scores for the symptomatic and asymptomatic infection; (+): positive and (-): negative

Simultaneous detection of *Cryptosporidium* and *Giardia* caused lower WHZ in 0-11 months of age; and lower HAZ and WAZ in 24-59 months of age among symptomatic MSD children in comparison to the *Cryptosporidium* and *Giardia-negative* children. Surprisingly, co-infection with *E. histolytica* and *Cryptosporidium*; and *Giardia* with *E. histolytica* had a protective effect on child growth in 0-11 months of age who were presented with diarrhea [supplementary table 5 (a)]. On the other hand, co-infection with *Giardia* and *E. histolytica* had lower WAZ and WHZ among 12-23 months old children compared to the *Giardia* and *E. histolytica* negative children [supplementary table 5 (b)].
