# Supplementary material for: Symptomatic and asymptomatic enteric protozoan parasitic infection and their association with subsequent growth parameters in under five children in South Asia and sub-Saharan Africa
Source: PLoS Negl Trop Dis. 2023 Oct 10;17(10):e0011687. doi: 10.1371/journal.pntd.0011687 (PMC10588856; doi:10.1371/journal.pntd.0011687)
Supplement: Attachment — Submitted filename: response letter_revised.docx [file pntd.0011687.s006.docx]

**Response to Reviewers’ comments:**

**Reviewer #1**:

-Are the objectives of the study clearly articulated with a clear testable hypothesis stated?
yes
-Is the study design appropriate to address the stated objectives?
Yes
-Is the population clearly described and appropriate for the hypothesis being tested?
Yes GEMS samples are taken for testing hypothesis of Giardia / parasite infection on growth faltering
-Is the sample size sufficient to ensure adequate power to address the hypothesis being tested?
7800 children's data from GEMS being used.
-Were correct statistical analysis used to support conclusions?
Yes appropriate for growth data
-Are there concerns about ethical or regulatory requirements being met?
Gems is a multi-site project and ownership of data is with PIs of site. It requires ERC exemption, or all site PIs should be informed.

**Response**: Thank you for your constructive feedback. The publicly available GEMS dataset was obtained from ClinEpiDB, an open-access data repository, and we have followed all ethical guidelines and regulations to access and utilize the data. Moreover, we have taken the additional step of seeking approval from GEMS site PIs via email to ensure that our work aligns with their expectations and respects their valuable contributions to the dataset. We mentioned this in the data availability statement of the manuscript. We also provided the link to ClinEpiDB here for clarification.

<https://clinepidb.org/ce/app/workspace/analyses/DS_841a9f5259/new/details#AccessRequest>

**Reviewer #2:** Authors have employed pathogen results detected by ELISA. What was the reasoning for not employing the detection results from the later reanalysis by molecular techniques? Would this not have given more accurate results, especially for the low-grade protracted infections that both Giardia and Cryptosporidium can cause? And thereby perhaps a better corrective balancing for all the co-pathogen variables in the regression model?

**Response:** We acknowledge that detecting pathogens through molecular techniques could potentially offer more accurate results, but this was outside the scope of this limited secondary analysis of existing data. The GEMS dataset we used in our study was obtained from ClinEpiDB, which is an open-access data repository, and the primary author does not have access to the stored samples. The data available on ClinEpiDB for Protozoa (*Cryptosporidium, E. histolytica,* and *Giardia*) was primarily detected using ELISA methods and as of now, data related to protozoa detection through molecular techniques is not available on ClinEpiDB. We are aware of the importance of molecular techniques in pathogen detection, and we hope that future updates to ClinEpiDB will include such valuable data. In the meantime, we will make the best use of the available data to draw meaningful insights. We also provided the link to ClinEpiDB here for clarification.

<https://clinepidb.org/ce/app/workspace/analyses/DS_841a9f5259/new/variables/EUPATH_0000609/EUPATH_0030117>

**Comment**: Anthropometric measures were done at two timepoints. It is commendable that both measures are available and included in the analysis, and that measures at enrolment were done after rehydration in case of MSD. This makes possible an evaluation of anthropometry status both at enrolment and at follow-up and could help solve the puzzle regarding the degree to which malnourished children more often acquire protozoan infections versus the hypothesis that protozoan infections cause growth faltering, and how these factors may interact.
Perhaps it is just my poor understanding of the statistical model, but I am bit confused about the results given in table 1 and 2. It is stated that they are “association between enteric protozoan parasites infection and child HAZ/LAZ, WAZ, and WHZ”. I then wonder; Are they expressing the deviation in score for each given protozoan pathogen from the expected increase in anthropometric scores in the whole cohort?
Authors also state that “Giardia infection was associated with lower HAZ (-0.13; 95% CI: -0.17, -0.09…». Does it actually mean a “lower than expected” or “reduced” HAZ score? Or the adjusted difference in the HAZ score from each child’s baseline 2 months previously, as if no growth was expected in that period?
I think the authors need to communicate clearer how the results convey the difference between enrollment and follow-up for children found positive for these pathogens. Please give a clear description of the metric you use so that also non-epidemiologists can understand it. In Nasrin et al 2022 the data is given as “Difference in ΔHAZ (95% CI)”. Is that what is actually meant?
I would also strongly recommend to provide an example of what the coefficients given actually indicate. For example; what does a HAZ coefficient of -0.13 mean in height loss in cm for a child aged one year?

**Response:** Allow us to provide further elaboration on the approach we took in our analysis. In Dr. Nasrin's article (Nasrin D, Blackwelder WC, Sommerfelt H, Wu Y, Farag TH, Panchalingam S, et al. Pathogens Associated with Linear Growth Faltering in Children with Diarrhea and Impact of Antibiotic Treatment: The Global Enteric Multicenter Study. J Infect Dis. 2021;224(Supplement_7): S848-S55. doi: 10.1093/infdis/jiab434), the primary outcome of interest was the change in linear growth, specifically the change in the Height-for-Age Z score (ΔHAZ), which was calculated for each child by measuring the difference in HAZ from enrollment to follow-up. The data for this change in ΔHAZ was presented with corresponding 95% confidence intervals (95% CI). In our study, we did not use the adjusted difference (Δ) in child Z scores. Instead, we utilized a Generalized Linear Model (GLM) for the final analysis. In our analysis, we did not compare the baseline anthropometry between symptomatic MSD (cases) and asymptomatic (control) children, rather separate models were performed to see the association of enteric protozoan parasites infection with a child’s z-scores for the symptomatic and asymptomatic infection. Hence ΔHAZ/ WAZ/ WHZ are the mean differences, and those cannot be used in multiple linear regression models. Since the data involved repeated measurements over time, we adjusted the time variable as a covariate. The anthropometric measurements were taken at two-time points: enrollment (after rehydration in MSD children) denoted as 0, and approximately day 60 during follow-up, denoted as 1. Our main focus was to examine the overall impact of protozoan-positive MSD and asymptomatic healthy children, not exclusively limited to malnourished children. By adjusting the “time” variable and incorporating the anthropometry (Z scores data) from baseline to endline, the results of the GLM model provided insights into changes in HAZ, WAZ, and WHZ scores which was expected in the follow-up in comparison with the children having stool negative for a protozoan. Specifically, for example, we observed that asymptomatic children who tested positive for *Giardia* at enrollment exhibited a lower HAZ score of 0.13 (with a coefficient of -0.13) which was lower than expected during the follow-up compared to children who were *Giardia* negative. In this context, our comparator group consisted of *Giardia*-negative children, enabling us to draw comparisons (Similar interpretation for all 3 parasites). Regarding your comment on the interpretation of the HAZ coefficient of -0.13, we appreciate the clarification. HAZ represents the height for age Z score, which is the standardized score for assessing a child's linear growth, not an indication of mean height. We acknowledge the importance of providing accurate explanations to ensure a clear understanding for our readers. In the result section we clarify the statements: “*Giardia* infection was associated with lower HAZ (-0.13; 95% CI: -0.17, -0.09) and WAZ (-0.07; 95% CI: -0.11, -0.04); but not WHZ (-0.02; 95% CI -0.06, 0.02) which was lower than expected in comparison to the Giardia negative children, these findings were also observed amongst the different age groups of children except for older children (24-59 months).” Similarly, “Cryptosporidium infection was associated with lower HAZ (-0.17; 95% CI: -0.23, -0.11), WAZ (-0.25; 95% CI: -0.31, -0.19), and WHZ (-0.23; 95% CI: -0.30, -0.17) (Table 1) which was lower than expected during ~60 days follow up compared to the Cryptosporidium negative children.” We are genuinely grateful for your constructive suggestion, and as a result, we have revised the results section to enhance clarity and facilitate a better comprehension of our study findings.

**Comment:** Important co-pathogens other than protozoans were included in the GLL model as co-variates, while presence of protozoans were explanatory variables. Can the authors clarify how co-infections with two or three protozoans (which are not uncommon) were handled by this model? For example, were any interactions between these explanatory variables tested? The Nasrin et al 2022 included a multiple-pathogen model estimate. Is that the same that was used in the present analyses?

**Response:** We have carefully considered your comments and re-evaluated our analysis to address the points you raised. For Tables 2 and 3, we utilized a multiple linear regression model with the dependent variables being HAZ/LAZ, WAZ, and WHZ. This model allowed us to explore the association between enteric protozoan parasite infections and a child's height-for-age, weight-for-age, and weight-for-height z-scores over time. We performed separate analyses for symptomatic and asymptomatic infections, adjusting for potential covariates including multiple co-pathogens to account for confounding factors. Indeed, the multiple regression model played a crucial role in clarifying the relationships between various variables, including co-infections/ co-pathogens. By considering multiple pathogens simultaneously, we were able to assess how different pathogens influenced the outcomes. Moreover, we investigated interaction effects in the model, this step allowed us to capture potential synergistic or antagonistic interactions between enteric pathogens, which could have implications for child growth.

In response to your concern about co-infections, we examined the data and found that only a small percentage, specifically 3.01% of children, tested positive for co-infections with more than one protozoan in our dataset. Although co-infections are essential to consider, the relatively low prevalence of these cases in our study population indicates that they may not have a significant impact on the overall results. To provide further clarity, we have included the results of the presence of more than one protozoan parasite in supplementary tables 3 and 4: **“**In supplementary tables 3 and 4 children having more than one enteric protozoan parasite infection and protozoan parasite co-infections detected among asymptomatic and symptomatic MSD children were presented. Only 3% of children had more than one parasite detected in stool during enrollment. Around 3% (n=662) children were positive for more than two parasites in stool and less than 0.05% (n=14) children were positive for three parasites simultaneously (Supplementary Table 3). Co-infection with *Cryptosporidium* and *Giardia* (8.7%); *Cryptosporidium* and *E. histolytica* (3.4%); *Giardia* and *E. histolytica* (2.5%) were more observed among the symptomatic MSD children (Supplementary Table 4 in comparison to asymptomatic children.” This addition will help readers gain a comprehensive understanding of the data and our findings. Additionally, we took the time to check for potential effect modifiers in the final model by checking the interactions. Initially, we checked the Mantel-Haenszel test to detect the heterogeneity of the association by different pathogens. We found no significant heterogeneity by enteric pathogens for the HAZ, WAZ, and WHZ associations. We also checked by using a likelihood-ratio test (LRT) and found that without the interaction term, gave a better fit to our model. After conducting this analysis, we found no significant effect modifiers among the explanatory variables (enteric pathogens and protozoan). We addressed this in the statistical analysis part: “Similar model was performed for the detection of the association of protozoan parasite co-pathogens and child growth parameters after adjusting for the potential covariates. We checked for potential effect modifiers in the final model by checking the interactions. Initially, we checked the Mantel-Haenszel test to detect the heterogeneity of the association by different pathogens. A likelihood-ratio test (LRT) was performed and found that without the interaction term, the model had a better fit.”

According to your suggestion, we re-analyze the data for the **co-infection with more than one parasitic protozoan in Table 4.** Along with that another supplementary table (S3 table) was attached for the Enteric protozoan parasite co-infections detected among asymptomatic and symptomatic MSD children in GEMS (n=22,567). For the age-stratified association supplementary table 5 (S5 (a) and 5 (b) tables) is also attached to the manuscript on Age-specific association between enteric protozoan parasites co-infections and child anthropometric measurements: results of multiple linear regression modeling (dependent variables— HAZ/LAZ, WAZ, and WHZ) among under 5 children in South Asia and sub-Saharan Africa both for symptomatic and asymptomatic children.

**Results**

**Reviewer #1**: -Does the analysis presented match the analysis plan?

Analyses plan is adequate.

-Are the results clearly and completely presented?

Yes
-Are the figures (Tables, Images) of sufficient quality for clarity?

Need to submit better-quality pictures

**Response:** Thank you for your encouraging comments. We have prepared the images for better quality accordingly.

**Reviewer #2:** Authors start by giving prevalences of protozoans at different sites. In supplementary table 1 and 2 are given the number of positives for each of the three protozoan pathogens. Obviously, there must have been many samples positive for more than one protozoan pathogen. How did the authors arrive at the numbers in these tables, that are without protozoan co-infections, but still add up to 100%? How were co-infections dealt with? This could be better explained in the methods chapter, and some measure of the overlap given.

**Response:** For better clarification of the supplementary tables 1 and 2, we have added the number of negatives for each of the protozoan pathogens along with the p-value by Chi-square and the proportion test was used to see the association between two categorical variables, and the t-test was used to see the mean difference between two groups for symmetric distribution. We mentioned this in the statistical analysis part also: “Chi-square and proportion test were used to see the association between two categorical variables and the t-test was used to see the mean difference between two groups for symmetric distribution.” **For the number and percentage of co-infections with more than one protozoan parasite, we added supplementary Tables 3 and 4**. In the result section we added “The baseline demographic characteristics of the children are presented in supplementary tables 1 and 2. For this analysis, we included 7800 children. Among For the asymptomatic children, Cryptosporidium was detected in 843 (18.3%), Giardia in 3470 (75.2%), and E. histolytica in 299 (6.5%). Cryptosporidium and E. histolytica were more often (> 40%) detected among younger children (0-11 months), and Giardia was frequently detected among the older children (more than two years old children) among whom, more than 40% were female. For the MSD cases, Cryptosporidium was detected in 1123 (35.2%), Giardia in 1786 (56%), and E. histolytica in 279 (8.8%). Around 55.6% of children in the 0-11 months age group were Cryptosporidium positive. About 40% of children were from the older age group who were Giardia positive. But E. histolytica was less common among the children who were 24 months old and above. Dysentery (42.3%) occurred substantially more frequently for the E. histolytica-positive children and fever (60.1%) were most observed in Cryptosporidium-positive and Giardia-positive children where 55.56% E. histolytica-positive children were presented with fever.”

**Comment:** Ideally anthropometry follow-up data should be given also for the co-infections as has been shown to give interesting results in a recent paper by Kabir et al. 2023 https://doi.org/10.3389/fnut.2022.1081833 As we know there are interactions here the present manuscript might miss interesting findings by seemingly presenting only single infection analysis.

**Response:** Kabir et al. 2023 showed very interesting results in their SEEM community-based intervention study along with anthropometric data collection on a monthly basis. However, in GEMS there are only 2 points of anthropometric data available. We adjusted the time variable in the final model considering 2 separate time points in a single time point for the z scores. As we mentioned earlier, initially, we checked the Mantel-Haenszel test to detect the heterogeneity of the association by pathogens. We found no significant heterogeneity by enteric pathogens for the HAZ, WAZ, and WHZ associations. We also checked by using a likelihood-ratio test (LRT) and found that without the interaction term, gave a better fit to our model. After conducting this analysis, we found no significant effect modifiers/interactions among the explanatory variables (enteric pathogens and protozoan). And mentioned it in the statistical analysis part: “Similar model was performed for the detection of the association of protozoan parasite co-pathogens and child growth parameters after adjusting for the potential covariates. We checked for potential effect modifiers in the final model by checking the interactions. Initially, we checked the Mantel-Haenszel test to detect the heterogeneity of the association by different pathogens. A likelihood-ratio test (LRT) was performed and found that without the interaction term, the model had a better fit.” We created a new variable considering the presence of more than one parasite. And observed that, only 2.9% of children were positive for two parasites (co-infection) and only 0.06% of children were positive for three parasites (co-infection). We used the same technique followed by a MAL-ED article by James A Platts-Mills et al.

(<https://www.ncbi.nlm.nih.gov/pmc/articles/PMC7328884/>). We prepared supplementary Tables 3 and 4 mentioning the co-infections with more than one parasite.

| **Stool (+) for parasites** | **n= 22566 (%)** |
| --- | --- |
| No parasite | 15456 (68.5) |
| At least a single parasite | 6434 (28.5) |
| At least two parasites | 662 (2.9) |
| Three Parasites | 14 (0.06) |

We have also re-analyzed the data for co-infections and added Table 4 and supplementary tables 5(a) and 5(b) for age-stratified association with child growth following the analysis plan shown in Kabir et al. 2023. We added the statement in the result section: “To gain further insight into the association of protozoan parasites detection with child growth, we explored the role of co-pathogen colonization. A significant agreement was observed between the presence of Giardia with the presence of Cryptosporidium on lower WAZ (β: -0.18; 95% CI: -0.31, -0.05; P= 0.01) and WHZ (β: -0.21; 95% CI: -0.35, -0.06; p <0.001); On the other hand, co-infection of Giardia and E. histolytica has a protective role as children with both the pathogens had higher growth than the expected HAZ (β: 0.32; 95% CI: 0.07, 0.58; p= 0.01) and WAZ (β: 0.30; 95% CI: 0.03, 0.56; p= <0.001) among the children exhibiting symptoms. No association was found regarding the co-colonization of Cryptosporidium with E. histolytica for the children having diarrhea (table 4).” and “Most co-infections did not reveal an association with growth measures (Table 4). For symptomatic MSD children, exceptions included negative associations with growth when children were positive for Cryptosporidium with E. Histolytica negative and Cryptosporidium regardless of Giardia status (i.e., both giardia positive and negative). These relationships with lower growth when children had co-infection with both Cryptosporidium and E. histolytica also held for asymptomatic MSD children, but among these children, negative associations with growth existed when children were positive for Giardia regardless of Cryptosporidium status.”

**Comment:** Figure 1 gives a good overview of the participants, and shows a low attrition rate for follow-up anthropometry measurement.

**Response:** Thank you for your encouraging comments.

**Comment:** Line 119: Were data from infants who were negative for protozoan parasites not included in the analysis? Would they not have provided important background data, especially for the asymptomatic group?

**Response:** We included the children who were positive for protozoan parasites and who were negative for protozoan parasites were also included in the analysis as the comparator group. We have rewritten the line according to your suggestion, “As the comparison group we used the baseline and endline follow-up anthropometry data for the E. histolytica, Giardia, and Cryptosporidium negative children.”

**Comment:** Baseline anthropometric data are given in supplementary tables 1 and 2 but not commented upon. Were there no significant baseline differences between protozoan pathogens? I think these results are also an important finding in such a large dataset, and should be stated, and possibly discussed later, as several studies to date have drawn (often uncertain) conclusions about malnutrition and protozoan infection based on such one-time measures. Asymptomatic controls seem to have higher WAZ and WHZ scores than symptomatic infants. I definitely would want a column showing the baseline data for the total population, including anthropometric data, in these two tables.

**Response:** Supplementary tables 1 and 2 are already modified according to your suggestion along with the significant baseline differences between protozoan pathogens. We also added Table 1 showing the baseline data for the total population, including anthropometric data for both the symptomatic and asymptomatic children as: “Baseline anthropometric measurements (WAZ mean ±SD: -1.08± 1.32 vs -1.51± 1.39 and WHZ: -0.47± 1.49 vs -1.06± 1.49) showed a statistical difference (p-value <0.001) between asymptomatic and symptomatic children respectively (Table 1). Symptomatic MSD children were more often (82.3%) using non-tube well water as the main source of drinking water compared to asymptomatic healthy children (82.3% vs 77.2%, p-value <0.001). Non-sanitary latrines were available for 6.4% of households. Cryptosporidium (11.9% vs 6.4%), E. histolytica (2.9% vs 2.3%), ETEC (11.3% vs 7.4%), rotavirus (18.5% vs 3.9%), and Shigella (11.8% vs 1.8%) were more frequently detected amongst the children exhibiting symptoms in comparison to asymptomatic children (p-value <0.001 for each pathogen) respectively, but Giardia was more isolated among the children without symptoms compared to symptomatic children (%%) (26.4% vs 18.9%, )p-value <0.001).

**Comment:** A short translation of the anthropometric measures into prevalence of MAM and SAM (based on WHZ) and stunting (based on HAZ) in the text would be helpful to put the study population nutritional status into perspective.

**Response:** We acknowledge that the individual measures of malnutrition (stunting and wasting: MAM/SAM) still hold value in certain contexts and can be helpful for communication and advocacy efforts, as they are more easily understood by policymakers and the general population. However, in our data presentation, we preferred Z scores over individual measures of malnutrition, in data analysis for several reasons making them a valuable tool for assessing and monitoring the nutritional status of under 5 children: firstly, Z scores are standardized measures that allow for easy comparison of nutritional status across populations and age groups. And most importantly, Z scores are continuous variables, providing more precise and detailed information about a child's nutritional status compared to categorical measures like stunting, wasting, and being underweight. This continuous nature enables us to capture subtle changes in nutritional status, which can be critical for early intervention and monitoring. Z scores are also sensitive to changes in nutritional status, making them suitable for tracking improvements or deteriorations in children's health over time. Z scores, however, consider the child's age, reducing the impact of random variations in individual measurements. Lastly, our objective is to identify children who are at risk of malnutrition (0-59 months) and provide valuable information for early intervention and targeted support for all the children positive for protozoan irrespective of malnutrition at enrollment. Moreover, we thought a single manuscript with lots of terminology (z scores and/ stunting, wasting) and analysis might be confusing and overwhelming for the readers.

**Conclusions**

**Reviewer #1**: -Are the conclusions supported by the data presented?
Yes
-Are the limitations of analysis clearly described?
Not mentioned
-Do the authors discuss how these data can be helpful to advance our understanding of the topic under study?
not really clear.
-Is public health relevance addressed?
Targeting enteric parasites in young children is mentioned

**Response:** We addressed the limitation of our analysis and the recommendations to advance our understanding of parasitic infections accordingly. The limitation of the study is stated as: “Our study was limited by the lack of data on maternal BMI, gestational age, birth weight, serum micronutrient levels, and enteric inflammatory biomarkers of the children. Additionally, only short-term impacts (60 days) were assessed, leaving open the possibility of subsequent catch-up growth. We could not detect the effect of the human immunodeficiency virus (HIV) as HIV data in the children’s population were not readily available for South Asia.”

How these data can be helpful to advance our understanding is stated as: “This enteric protozoan parasitic infection data can be highly valuable in advancing our understanding of enteric infections and their impact on child health and development. Our analysis includes information on co-infections and co-pathogens, providing insights into how multiple parasites or pathogens interact and influence each other's effects on child health. Understanding these interactions can have implications for diagnosis, treatment, and public health strategies. Our analysis findings can be used to assess the effectiveness of interventions targeting parasitic infections, such as WASH improvements, deworming programs, and nutrition interventions.”

**Reviewer #2:** The discussion needs to touch upon repeated results are brought forward here and how they relate to the previous publication by Nasrin et al 2022. For example, were Cryptosporidium in the symptomatic infants was published in that paper.

**Response:** We addressed this issue in the discussion section accordingly. “*Cryptosporidium* was associated with a greater decline in linear growth in toddlers with MSD, corroborating and expanding previous studies suggesting that *Cryptosporidium* in infancy imparts a lasting adverse effect on linear growth, including less weight gain in the first month of infection [9].”

**Comment:** It would be good to include the reasoning for analyzing single pathogens only, and not include results for protozoan co-infections. The authors have instead given estimates for “Presence of any one parasite” which I think gives less clinically interesting information. Please discuss the presence of protozoan co-infections in LMIC and preferably also include such co-infection analyses in this manuscript.

**Response:** We addressed these issues in detail accordingly in the result section as: “To gain further insight into the association of protozoan parasite detection with child growth, we explored the role of co-pathogen colonization. We observed an association (p= 0.01) and WHZ (β: -0.21; 95% CI: -0.35, -0.06; p <0.001); on the other hand, co-infection with both Giardia and E. histolytica was associated with higher growth than the expected HAZ (β: 0.32; 95% CI: 0.07, 0.58; p= 0.01) and WAZ (β: 0.30; 95% CI: 0.03, 0.56; p= <0.001) among the children exhibiting symptoms. No association was found between the co-colonization of Cryptosporidium and E. histolytica on diarrhea symptoms (table 4). We report on the prevalence of multiple infections in supplemental table 3 and compared enteric protozoan parasite co-infections between asymptomatic and symptomatic MSD children in supplemental table 4.” and discussion section on parasitic co-infections as: “Based on the analysis of data regarding single pathogen colonization, contradictory findings have emerged regarding the potential protective role of Giardia, particularly during early childhood [16, 26]. In animal model experiments utilizing protein-deficient mice, it has been observed that microbial adaptations to undernutrition, in conjunction with the cumulative impact of entomopathogens, significantly influence host growth, gut immune response, and metabolism [33]. In the context of Giardia and enteroaggregative Escherichia coli co-colonization, it has been observed that Giardia can effectively evade microbiota-mediated pathogen clearance under conditions of protein malnutrition. This evasion mechanism leads to the promotion of growth impairment and significant alterations in the abundance of small intestinal 16S, as well as mucosal immunity. These changes converge with the metabolic responses to exacerbate host growth impairment. [34]. Their data model the effect of early-life cumulative enteropathogen exposures on the disruption of intestinal immunity and host metabolism during crucial developmental periods [34]. We observed similar trends in our study of children where Cryptosporidium detection in the presence of Giardia had a negative impact on the child's growth, commonly among 24-59 months old diarrhoeal children. Similarly, simultaneously positive for Giardia and E. histolytica had poor growth in 12-23 months old children who were asymptomatic. As intestinal microbiota develops during the early months of life, this protection might provide the critical window of susceptibility. However, Cryptosporidium causes persistent diarrhea in young children in the LMICs. Although it has been suggested that the effect of infections on nutrition is usually transient because of catch-up growth, another community study from West Africa suggested that cryptosporidiosis in infancy has a permanent effect on growth [35]. Hence, further studies are required on the role of gut microbiota and enteropathogen co-colonization.”

**Comment:** The authors refer to several studies of growth faltering in Giardia and Cryptosporidium. I think there are at least two studies that also should be included in this discussion, the early study by Mølbak et al and the recent study by Kapir et al.

Mølbak K, Andersen M, Aaby P, Højlyng N, Jakobsen M, Sodemann M, et al. Cryptosporidium infection in infancy as a cause of malnutrition: a community study from Guinea-Bissau, west Africa. Am J Clin Nutr. 1997; 65(1):149–52. https://doi.org/10.1093/ajcn/65.1.149 PMID: 8988927

Kabir F et al. Impact of enteropathogens on faltering growth in a resource-limited setting. Front Nutr. 2023 Jan 10;9:1081833. doi: 10.3389/fnut.2022.1081833. PMID: 36704796

**Response:** We now mention the updated information from the above-mentioned two articles in our discussion section as: “Based on the analysis of data regarding single pathogen colonization, contradictory findings have emerged regarding the potential protective role of Giardia, particularly during early childhood [16, 26]. In animal model experiments utilizing protein-deficient mice, it has been observed that microbial adaptations to undernutrition, in conjunction with the cumulative impact of entomopathogens, significantly influence host growth, gut immune response, and metabolism [33]. In the context of Giardia and enteroaggregative Escherichia coli co-colonization, it has been observed that Giardia can effectively evade microbiota-mediated pathogen clearance under conditions of protein malnutrition. This evasion mechanism leads to the promotion of growth impairment and significant alterations in the abundance of small intestinal 16S, as well as mucosal immunity. These changes converge with the metabolic responses to exacerbate host growth impairment. [34]. Their data model the effect of early-life cumulative enteropathogen exposures on the disruption of intestinal immunity and host metabolism during crucial developmental periods [34]. We observed similar trends in our study of children where Cryptosporidium detection in the presence of Giardia had a negative impact on the child's growth, commonly among 24-59 months old diarrhoeal children. Similarly, simultaneously positive for Giardia and E. histolytica had poor growth in 12-23 months old children who were asymptomatic. As intestinal microbiota develops during the early months of life, this protection might provide the critical window of susceptibility. However, Cryptosporidium causes persistent diarrhea in young children in the LMICs. Although it has been suggested that the effect of infections on nutrition is usually transient because of catch-up growth, another community study from West Africa suggested that cryptosporidiosis in infancy has a permanent effect on growth [35]. Hence, further studies are required on the role of gut microbiota and enteropathogen co-colonization.”

**Comments:** Arguments relating to dysentery in lines 314 and onwards would be better placed in the section discussing E.histolytica. I think here the authors may also consider mentioning the effect of the different intestinal segments affected by each protozoan infection to explain the growth faltering seen in Giardia and Crypto, but not in E.histolytica. G and C infect the small intestine with its nutrient absorption role, while E.h is mainly a colonic pathogen.

**Response:** We modified the discussion section according to your suggestion as: “In a study of under 2 children from GEMS, dysentery was more likely to be treated with antibiotics than other enteric infections, potentially contributing to a growth-promoting effect on length and weight, although the impact of antibiotic treatment on growth in children with enteric infections remains unclear [30]. Moreover, Giardia and Cryptosporidium colonize the duodenum, jejunum, and ileum and are the most common sites for nutrients absorption; but E. histolytica colonizes the colon not in the small intestine [32], this might be another provable explanation of our findings.”

**Editorial and Data Presentation Modifications?**

**Reviewer #1:** This is a secondary analyses of primary GEMS data. It has been published elsewhere in detail, however this is a new finding of Giardia being negatively associated in asymptomatic children. While previously Giardia has shown to be protective in linear growth faltering. The definition of asymptomatic /controls should be clearly defined. Giardia was measured through ELISA and not through microscopy. q-RT PCR data is also available for GEMS pathogens, but it was not included in this manuscript. It is important to understand how data was accessed for this manuscript from all sites, for instance Pakistan Gems data was used but there is no author on this manuscript. If it is a longitudinal data set which is not clearly mentioned here then probably Mixed effects model is more appropriate. For secondary data analyses, I would use robust analyses plan that has not been previously used rather than simple regression analyses. Outcome measure is not clearly defined, the impact of entomopathogen was observed within two months of life, this time frame is very short to observe long-term effects of pathogen on growth.

**Response:** We appreciate your perspective, and we acknowledge that detecting pathogens through molecular techniques could potentially offer more accurate results. However, we would like to clarify that the GEMS dataset we used in our study was obtained from ClinEpiDB, which is an open-access data repository. Regrettably, the data available on ClinEpiDB for Protozoa (*Cryptosporidium, E. histolytica,* and *Giardia*) was primarily detected using ELISA methods. As of now, data related to protozoa detection through molecular techniques is not available on ClinEpiDB. We are aware of the importance of molecular techniques in pathogen detection, and we hope that future updates to ClinEpiDB will include such valuable data. In the meantime, we will make the best use of the available data to draw meaningful insights (we provided the screenshot for better clarification).

<https://clinepidb.org/ce/app/workspace/analyses/DS_841a9f5259/new/details>

After officially applying, the publicly available GEMS dataset was obtained from ClinEpiDB, an open-access data repository upon the approval of all seven site PIs, and we have followed all ethical guidelines and regulations to access and utilize the data responsibly. We mentioned this in the data availability statement of the manuscript. Moreover, we have taken the additional step of seeking approval from GEMS's seven site PIs via email to ensure that our work aligns with their expectations and respects their valuable contributions to the dataset. We mentioned the name of the co-authors from whom we got email approval regarding our manuscript.

Regarding the data analysis plan, we appreciate your suggestion of making a mixed-effects model. As per our understanding, the mixed effect model is more appropriate in RCT and Cohort studies. But in GEMS the anthropometric measurements were taken at two time points: enrollment (after rehydration in MSD children) denoted as 0, and approximately day 60 during follow-up, denoted as 1. By adjusting the “time” variable and incorporating the anthropometry (Z scores data) from baseline to endline, the results of the generalized linear model (GLM model) provided insights into changes in HAZ, WAZ, and WHZ scores which was expected in the follow-up in comparison with the children having pathogen negative in a single time point. So inter-cluster correlation was not possible here. Keeping in mind the possibility of the correlation among the seven sites, in the final model we adjusted the “site” variable to minimize the effect on the outcome variables.

We revised the outcome measures in the methodology section accordingly. The impact of entomopathogen was observed within two months of life, this time frame is very short to observe the long-term effects of the pathogen on growth. It is one of the major limitations of the study. We mentioned this in the limitation section.

**Reviewer #2:** Some Minor specific points
Line 34 in abstract, missing “-“ before 0.09 in the confidence interval.
Line 93-95: Incomprehensible sentence, please revise.
Line 197: The formulation “More than 55.6% of the …” seems a bit odd. Was it 55.6% or a unspecified higher percentage?
Line 240 Remove extra d in “investigated”
Line 247 reomve extra “both”

**Response:** We have addressed all those issues accordingly in the manuscript. For example, “Around 55.6% of children in the 0-11 months age group were Cryptosporidium positive.”; “HAZ [β: -0.13; 95% CI: ­-0.17, -0.09; p<0.001]”;

“If the risk of child malnutrition irrespective of diarrhea symptoms from different enteric protozoan parasites on child health is not equal, intervention programs aimed at improving nutritional status may not have the varying potential for reducing child morbidity and mortality.”

**Summary and General Comments**

**Reviewer #1**: 1- title should be modified - may consider omitting "young"
2- under 5 should be changed to "five"
3- in abstract add p value with B estimates
4- Older children term was used. please define "older"
5- line # 189 " this needs further elaboration if asymptomatic were healthy children?"
6- (+) sign should be written as positive
7- In figure 3 title healthy term was used. it is confusing whether asymptomatic were healthy as well.
8- Title of table should be revised. use anthropometric measurement rather than WHZ/LAZ/WAZ
9- Use either of LAZ or HAZ
10-Line # 242: Typo error: WHZ instead of HAZ
11- Figure 1: Gems control was mentioned but in whole manuscript control term was not used

**Response:** We revised the title and abstract accordingly. Title as: “Symptomatic and asymptomatic enteric protozoan parasitic infection and their association with subsequent growth parameters in under five children in South Asia and sub-Saharan Africa”. We defined the older children in the result section as (12-23 months and 24 to 59 months). In GEMS healthy children who were asymptomatic were enrolled as control children. We elaborated on this in the manuscript along with Fig 3 as “Fig 3. Site-Specific percentage of enteric protozoan parasites (Cryptosporidium, Giardia, and Entamoeba histolytica) isolated from the stool of asymptomatic under 5 children (healthy children enrolled as control from the community)”. Title of the tables are also revised: “Table 21. Among symptomatic children: association between enteric protozoan parasites infection and child anthropometric measurements: results of multiple linear regression modeling (dependent variables— HAZ/LAZ, WAZ, and WHZ) among the different age groups”. As GEMS enrolled 0-59 months old children. For the under two years old children during anthropometric measurement length was measured and for the 24-59 months old children who can stand, height was measured, for this reason, we used LAZ/HAZ. We addressed the typo error. Control children were the asymptomatic children enrolled as a healthy control in GEMS. As we are not focusing on comparing the case and control, we used the term “asymptomatic healthy children” in the manuscript.

**Reviewer #2:** The authors have examined a publicly available dataset from the GEMS study with regard to intestinal protozoans in symptomatic and asymptomatic infants below 5 years of age, and their association with anthropometric outcomes. They find significant associations between growth parameters and asymptomatic Giardia infection and for both asymptomatic and symptomatic Cryptosporidium infection, but not with E. histolytica. It is a valuable analysis and needed analysis of available data.

**Response:** Thank you for your valuable comments. We appreciate your esteemed expertise and feedback as a respected reviewer for our manuscript.

**Comment:** I have two major comments. How were protozoan co-infections dealt within the analysis and can the data say more about these? The other relates to clarity and communication of what the results actually mean. I think there is potential for a much clearer and better analysis and communication of these data. Structure could be improved by consistently starting with either symptomatic of asymptomatic infection in all sections of the manuscript.

**Response:** According to your suggestion, we re-analyzed the data for protozoan co-infection in Table 4 and Supplementary Tables 4 (a) and (b). We added the description in the result section and as well as in the discussion section. And changed the sentence structure as suggested.

**Comment:** I would recommend authors to put more emphasis on the fact that data presented here are including a post-infection follow-up period, which is very valuable. I suggest they bring it into the title so that it will be easily understood what this paper is about. For example “ … association with subsequent growth parameters in children below 5 years …” not just “… association with growth in young under 5 children …”

**Response:** We incorporated your advice in the title “Symptomatic and asymptomatic enteric protozoan parasitic infection and their association with subsequent growth parameters in children under five in South Asia and sub-Saharan Africa”.

**Comment:** In the introduction, please make a clear and comprehensive delineation of what was reported in the previous publication by Nasrin et al 2022 regarding HAZ in MSD cases in the GEMS data, and what new analyses are to be made in the present analysis.

**Response:** It was already mentioned in the Introduction section (lines: 78-81) as “In the Global Enteric Multicenter Study (GEMS) across seven global study sites, the presence of *Cryptosporidium* was significantly associated with a greater decline in linear growth than in those without *Cryptosporidium* [9], but no analysis has been conducted on asymptomatic children who were positive for intestinal protozoan parasites.”
